# Earthquake Probability Assessment for the Indian Subcontinent Using Deep Learning

**DOI:** 10.3390/s20164369

**Published:** 2020-08-05

**Authors:** Ratiranjan Jena, Biswajeet Pradhan, Abdullah Al-Amri, Chang Wook Lee, Hyuck-jin Park

**Affiliations:** 1 Center for Advanced Modeling and Geospatial Information Systems (CAMGIS), Faculty of Engineering and Information Technology, University of Technology Sydney, Sydney 2007, Australia; Ratiranjan.Jena@uts.edu.au; 2 Department of Energy and Mineral Resources Engineering, Sejong University, Choongmu-gwan, 209, Neungdong-ro Gwangin-gu, Seoul 05006, Korea; hjpark@sejong.edu kr; 3 Department of Geology and Geophysics, College of Science, King Saud University, P.O. Box 2455, Riyadh 11451, Saudi Arabia; amsamri@ksu.edu.sa; 4 Division of Science Education, Kangwon National University, Kangwondaehak-gil, Chuncheon-si, Gangwon-do 24341, Korea

**Keywords:** deep learning, GIS, earthquake probability, Indian subcontinent

## Abstract

Earthquake prediction is a popular topic among earth scientists; however, this task is challenging and exhibits uncertainty therefore, probability assessment is indispensable in the current period. During the last decades, the volume of seismic data has increased exponentially, adding scalability issues to probability assessment models. Several machine learning methods, such as deep learning, have been applied to large-scale images, video, and text processing; however, they have been rarely utilized in earthquake probability assessment. Therefore, the present research leveraged advances in deep learning techniques to generate scalable earthquake probability mapping. To achieve this objective, this research used a convolutional neural network (CNN). Nine indicators, namely, proximity to faults, fault density, lithology with an amplification factor value, slope angle, elevation, magnitude density, epicenter density, distance from the epicenter, and peak ground acceleration (PGA) density, served as inputs. Meanwhile, 0 and 1 were used as outputs corresponding to non-earthquake and earthquake parameters, respectively. The proposed classification model was tested at the country level on datasets gathered to update the probability map for the Indian subcontinent using statistical measures, such as overall accuracy (OA), F1 score, recall, and precision. The OA values of the model based on the training and testing datasets were 96% and 92%, respectively. The proposed model also achieved precision, recall, and F1 score values of 0.88, 0.99, and 0.93, respectively, for the positive (earthquake) class based on the testing dataset. The model predicted two classes and observed very-high (712,375 km^2^) and high probability (591,240.5 km^2^) areas consisting of 19.8% and 16.43% of the abovementioned zones, respectively. Results indicated that the proposed model is superior to the traditional methods for earthquake probability assessment in terms of accuracy. Aside from facilitating the prediction of the pixel values for probability assessment, the proposed model can also help urban-planners and disaster managers make appropriate decisions regarding future plans and earthquake management.

## 1. Introduction

**Figure 1:**
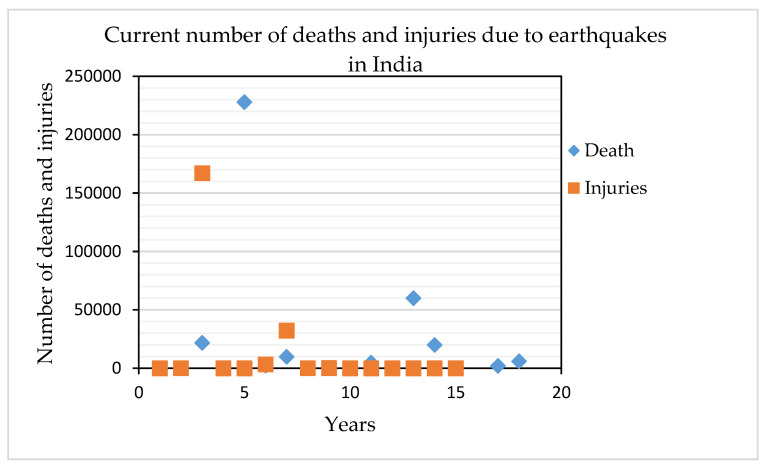
Statistics showing the deaths and injuries caused by earthquakes in India since 2000.

Earthquakes are devastating natural disasters that can cause mass destruction and result in the loss of properties and human lives. Therefore, earthquake prevention, prediction and probability monitoring are essential, along with hazard, risk and mitigation preparation. Countries, such as China, Taiwan, Japan, USA and Chile have developed early warning techniques and are highly advanced in earthquake research. However, traditional approaches are not used in the recent hazard assessment or monitoring of earthquakes because these methods are unable to achieve good results [1,2]. Waveform autocorrelation is a popular method for earthquake prediction from seismogram records [3]; meanwhile, deep learning is the latest technique for both earthquake prediction and probability assessment. However, some precursors can also be used for the probability and prediction of earthquakes. Seismological gravity monitoring was adopted in China after the occurrence of the 7.2 magnitude Xingtai earthquake in 1966 [4]. A mobile gravity survey that satisfactorily recorded gravity readings was conducted to measure gravity near the earthquake epicenter. Therefore, earthquakes can be predicted on the basis of precursors through recent knowledge of gravity and magnetic changes in the earthquake-prone areas [5]. Some major earthquakes in the world have occurred in India, and the development in earthquake study has started early in this country [6]. Therefore, India is currently developing seismic codes, code-compliant, and earthquake-resistant structures, institutional development, education, training, manpower development, and necessary research facilities [7]. India had experienced a huge number of earthquakes and the updated total deaths and injuries due to earthquake events are presented in Figure 1.

### 1.1. Global Earthquake Probability Assessment

Krinitzsky [8] calculated probabilistic earthquake ground motions by implementing the Gutenberg-Richter magnitude and recurrence relationship. However, several assumptions in probabilistic seismic hazard analysis were considered during the analysis. Therefore, though it is needed to make some essential assumptions in probability theory, it nevertheless has limited validity that leads to unsatisfactory results for critical structures. Hardebeck [9] mentioned that in quantitative earthquake probability assessment, the main concepts must be incorporated such as stress triggering and fault interaction. He introduced a novel method for translating to earthquake probability from stress changes. The proposed method could be potentially used with fault models. He concluded that stress change calculations would be useful for low slip-rate faults only in the long-term assessment. Nevertheless, stress-triggering calculations are generally applied in the short-term followed by a major earthquake. Parsons [10] pointed out that earthquake rate changes due to stress change. However, he answered some questions such as earthquake probability changed due to stress change and data used to generate parameters for forecasting. According to his study, a fault system with an understandable history of earthquakes, a ratio of stress change to stressing rate should be a minimum of 10:1 to 20:1 that can skew probabilities with >80–85% confidence level. Shapiro et al. [11] investigated the distribution of event magnitudes and described that with constant injection pressure, the probability of a magnitude >4 Mw could originate. They analyzed the injection time in bi-logarithmical law with a coefficient of proportionality. They observed that the diffusion process of pressure diffusion obeying a Gutenberg-Richter relationship in a poroelastic system with sub-critical cracks which were randomly distributed well explains the observations. Hagiwara [12] estimated the large-scale earthquake probability geodetically from crustal strain in a seismic prone area. He implemented the Weibull distribution function, generally used for research on quality control. Therefore, it was implemented in this study for probabilistic crustal strain treatments. Shcherbakov et al., [13] described that unexpected occurrence of earthquakes could trigger subsequent events that can lead up to powerful earthquakes. They developed a methodology to compute the extreme earthquake probabilities to be above some specific magnitudes. Authors combined the extreme value theory with the Bayesian method, having an assumption of the Epidemic Type Aftershock Sequence process. They applied and analyzed the proposed methodology to the 2016 Kumamoto earthquake sequence in Japan. Their study might help to find out the probabilities in several stages of an earthquake sequence for large events. Brinkman et al. [14] described that for mitigation purposes, there is a requirement of forecasting seismic events. Authors in this study present a simple earthquake model to investigate if the correlation between small earthquakes and tidal stresses could inform about the probability of large events. The model shows that the significant correlations between periodic stresses and low magnitude events could lead to large earthquakes. The recent observations agreed with the obtained results. It is expected that important input parameters and fault-specific information in the model could provide new tools in achieving the improved probabilities of large earthquakes. Wyss and Wiemer [15] described in their study that the landers event redistributed the stress in southern California, shutting off the earthquake in some locations while leading to increasing seismicity in neighboring locations until date. In more recent work, Jena et al. [16] developed an integrated model of artificial neural network-analytical hierarchy process (ANN-AHP) for earthquake risk assessment and applied it to the Aceh province in Indonesia. They observed that the southwest part of the city is highly probable for earthquakes based on the ANN technique with an accuracy of 84%.

### 1.2. Probabilistic Earthquake Hazard Assessment in India

Parvez and Ram [17] estimated the probability of recurrence of large events with magnitude greater than 7.0 based on four probabilistic models such as Gamma, Weibull, Lognormal, and Exponential in NE India. The parameters were estimated using the Method of Moments (MOM) and Maximum Likelihood Estimates (MLE). The estimated cumulative probability ranges from 0.881 to 0.995 for 40 years from 1964 until 1995. The conditional probability was estimated and it was expected a great earthquake would hit NE Indian peninsula at any time in the future. Tripathi [18] conducted a probabilistic study on the occurrence of large earthquakes (Mw ≥ 6.0 and Mw ≥ 5.0) using models such as Weibull, Gamma, and Lognormal. The 180 years of earthquake catalog having magnitudes ≥5.0 Mw used in this study. However, MLE was applied for earthquake hazard parameters. For events (Mw ≥ 5.0), the cumulative probability estimated as 0.8 based on Lognormal and Gamma models while it is 0.9 based on the Weibull model. However, for Mw ≥ 6.0, the probability is 0.8 while it is 0.9 in the next 53, 54, and 55 years based on Weibull, Gamma, and Lognormal models, respectively. Yadav et al. [19] worked on the probabilistic recurrence of earthquakes in Northeast India. Wei-bull, Gamm, and Lognormal models were implemented along with the earthquake catalog spanning the period from 1846 to 1995. The resulted cumulative probability for a large earthquake of magnitude 7.0 can be reached to 0.8 after about 15–16 years and 0.9 after about 18–20 years from the time of the last earthquake (1995) in the study area. Thaker et al. [20] performed the probabilistic seismic hazard analyses in the Surat region, where five seismotectonic sources were considered. The results obtained from the study focus on bedrock level peak ground acceleration (PGA) and response spectra using the local site effects. The values of PGA and spectral acceleration observed as 0.01 s and 1.0 s whereas the calculated probability of exceedances is 10% and 2% in 50 years. Gupta and Singh [21] examined earthquake occurrences before 30 December 1984 and the earthquake on 6 August 1988. They considered seismicity examples and applied the preparatory swarm theory of Evison [22] to develop a prognosis. Four micro-earthquake overviews were presented from 1983 to 1986 through an impermanent five-station system in various parts of the Shillong Plateau and neighboring regions [23,24]. The chronological change in the miniaturized scale of the seismicity rate was examined. Sitharam et al. [25] estimated the spatial variation of the spectral and peak horizontal acceleration (PHA) values in South India. Sitharam et al. [26] also studied probabilistic seismic hazard assessment in India using a topographic gradient.

In the current study, a convolutional neural network (CNN) model was developed for earthquake probability assessment. To date, no study has been conducted using deep learning and GIS for the earthquake probability assessment. However, this model was implemented for the Indian subcontinent for the first time. The current study aims to update the earthquake probability map of the Indian subcontinent. Some researchers have conducted probabilistic seismic hazard mapping in India. However, they have used traditional methods that exhibit many uncertainties. As the Earth’s internal structure is complicated, therefore traditional models consider some assumptions such as (a) considering segments of a specific fault as one based on uniform seismicity, (b) local attenuation equation to calculate PGA (c) earthquakes occur randomly through time and space. However, these assumptions were not considered in the current study, which could lead to uncertainties on data and accuracy of results. Therefore, mapping the probability of earthquakes and updating old maps are essential. The specific objectives of this study are as follows: (1) to develop a CNN model for prediction of earthquake classification and (2) to develop a probability map based on the CNN prediction of classification results. The model will enable performance accuracy evaluation and loss estimation. The proposed model was tested in India and covered nine probability factors. The estimation result was analyzed by calculating and comparing area and length with those of published old seismic hazard maps.

## 2. Seismic Tectonics of the Study Area

### Study Region

**Figure 2:**
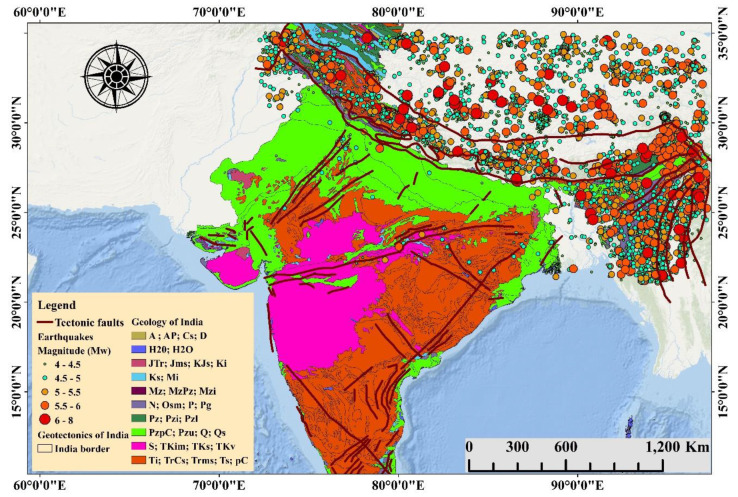
Location of the study area showing the tectonics and detailed geology of the Indian subcontinent with earthquake events of Mw > 4 in the Himalayan region (events and faults information were collected from articles [27,28,29]). (A: Archean, Ap: Archean and Proterozoic, Cmsm: Cambrian sedimentary and metamorphic rocks, Cs: Carboniferious sedimentary rocks, D: Devonian rocks, H20: other regions, JTr: Triassic and Jurassic rocks, Jms: Jurassic metamorphic and sedimentary rocks, Jks: Jurassic and Cretaceous sedimentary rocks, Ks: Cretaceous sedimentary rocks, MzPz: Paleozoic and Mesozoic metamorphic rocks, Mzi: Mesozoic intrusive rocks, N: Neogene sedimentary rocks, Osm: Ordovician metamorphic and sedimentary rocks, Pg: Paleogene sedimentary rocks, Pr: Permian rocks, Pz: undifferentiated Paleozoic rocks, Pzi: Paleozoic igneous rocks, Pzl: Lower Paleozoic rocks, Pzu: Upper Paleozoic metamorphic rocks, PzPc: Paleozoic undivided Precambrian rocks, Q: Quaternary sediments, Qs: Quaternary sand, S: Silurian rocks, TKim: Cretaceous and Tertiary igneous and metamorphic rocks, TKs: Cretaceous and Tertiary sedimentary rocks, TKv: Cretaceous and Tertiary volcanic rocks, Ti: Tertiary igneous rocks, TrCs: Upper Caboniferious–Lower Triassic sedimentary rocks, Tims: Triassic igneous and sedimentary rocks, Ts: Tertiary sedimentary rocks, and Pc: Precambrian rocks).

The Himalayas is located in the northern part of the Indian subcontinent, which is seismically dynamic [26]. The study area is situated at a latitude and longitude of 20.5937° N and 78.9629° E based on the WGS 84 geodetic coordinates system, respectively (Figure 2). This area is the seventh-largest nation by territory, the second most populated country in the world. The total population in India is 1,324,171,354, and the total country area is 3,287,263 km^2^ [27]. The most common rock types in the Himalayan Mountains are oceanic and ophiolite rocks. In the past decade, four extraordinary seismic tremors (M > 8) occurred along the Himalayas, along with countless moderate-sized quakes. Nearby seismic systems are confined toward the western Himalayas and the Assam area. Hence, seismic tremor hypocenters for the Himalayan earthquakes depend on the teleseismic areas. Nevertheless, the focal depths of these quakes can exhibit errors of up to 50 km. The distortion of the Himalayas is due to the gravitational distribution in the area. The main boundary thrust is the present active fault system of the Himalayan arc based on data recorded by teleseismic earthquakes. Medium-sized thrust-type quakes suggest that numerous chances of great Himalayan earthquakes can occur along the same detachment region.

## 3. Geopotential Data Acquisition and Analysis

### 3.1. Catalog

Kolathayar et al. [28] developed an earthquake catalog for India and its adjoining regions. This catalog comprises data from the literature and different national and international agencies. The aforementioned authors designed relationships that combine different extent scales and standardized the catalog in a combined moment extent scale. In an earthquake study, using only a complete catalog is insufficient in probability mapping. However, data from single or multiple sources can be collected. Several public and private organizations provide open access earthquake catalogs for research purposes. These accessible sources include the United States Geological Survey (USGS), Advanced National Seismic System (ANSS), Northern California Earthquake Data Center (NCEDC), and the National Earthquake Information Center (NEIC). The catalog of Kolathayar et al. [28] contains information collected from USGS, with specifications of magnitudes, depth, and location with coordinates of latitude between 9.269° N and 37.435° N and longitude between 66.533° E and 97.822° E based on the WGS 84 geodetic coordinates reference system. This distributed earthquake catalog and data from USGS were used to train the model, predict classifications, and generate the probability map in the present study. 

### 3.2. Local Sources

Local sources refer to the raw data sources in India used in this study. The Geological Survey of India published the seismotectonic atlas [29], which is regarded as an authentic referral for seismic source identification. Iyengar and Ghosh [30] conducted studies in Delhi and produced a micro-zonation map of earthquake hazards. Nath et al. [31] performed micro-zonation in Sikkim as a case study. Boominathan et al. [32] conducted earthquake hazard mapping in Chennai using local site effects. Kanth and Iyengar [33] studied seismic hazards in Mumbai. Anbazhagan et al. [34] and Vipin et al. [35] researched seismic probability in Bangalore and South India, respectively. Detailed information about lineaments, faults, shear zones, and several features in India was documented by Narula et al. [29]. In the present study, a detailed geological map representing earthquake hazards in India and showing faults, thrusts, and earthquakes stronger than 4 Mw was used to generate several thematic layers and implemented in CNN model [36,37]. This information, along with lithology information, was processed in the present study through the digitization and georeferencing of available maps using ArcGIS 10.4. and the clustered earthquake information. The final map superimposed all the information prepared in thematic layers. Table 1 summarizes the list of input paramters and their sources.

### 3.3. Thematic Layers

**Figure A1:**
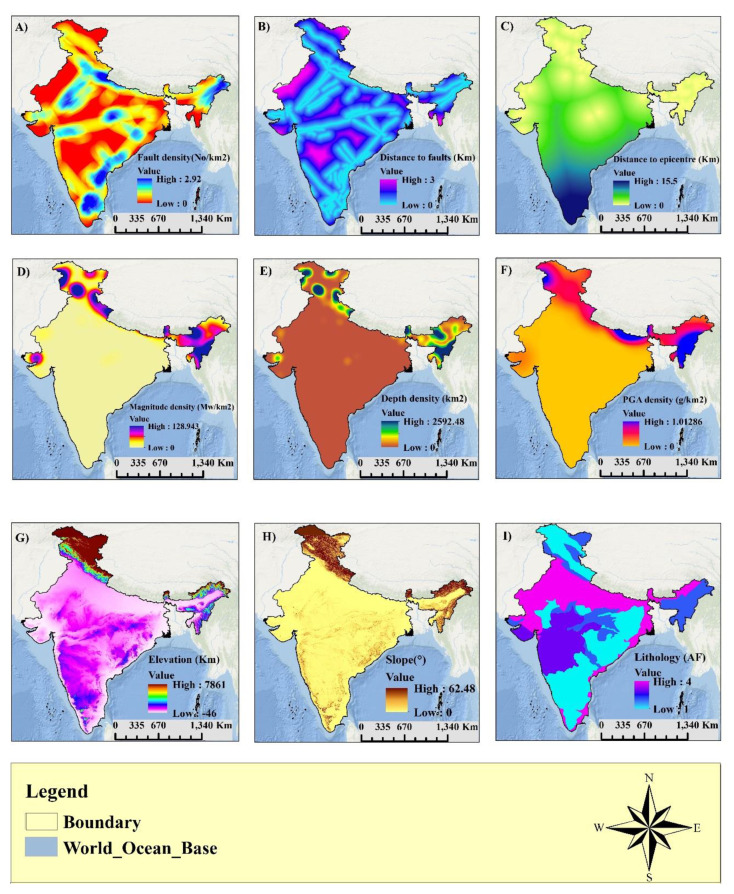
Thematic data indicators for the earthquake probability assessment. (No.: number; km: kilometer; km^2^: square kilometer; °: degree; g: gal).

In this study, various thematic layers (Figure A1) were produced after an extensive review of the literature [38]. An underlying process is linked to an analytic framework to construct a map using a geographic information system (GIS). The data used in this study does not support choropleth maps. Cartogram and chorochromatic maps were produced using statistical software, MS Excel, and ArcGIS (http://www.pbcgis.com/normalize/). Probability assessment is challenging without selecting appropriate criteria on which the model fully depends upon. To achieve good accuracy in producing a probability map, the most essential and useful steps are selecting important indicators and removing noise and heterogeneity from the data. In this study, freely available 30 m resolution digital elevation model (DEM) data was used to generate several layers. The UTM/WGS 1998 reference system was used to map and produce thematic layers. The following thematic layers were derived from several sources: Euclidean distance from normal, strike-slip and thrust faults (including major photo lineaments), fault density, lithology with an amplification factor value, slope angle map, elevation, magnitude density, epicenter distance, depth density, distance from the epicenter, and PGA density [39,40,41,42]. The layers were presented in a stretched format starting from negative values to positive values. Table 1 provides an overview of the importance of data layer preparation. These layers can be understood by uncovering concepts, emerging patterns, themes of objectives, and insight logic. Pre-processing was performed on the basis of the literature [30]. Subsequently, all the layers were converted into multiple values by points using GIS tools. All the values for each pixel were produced in an Excel file. Next, some negative and illogical values were removed because they can result in noise. Post-processing was performed after the prediction task was completed for the testing dataset [38]. The proposed model predicted the exact values of each pixel. The values were added to the GIS database, and a point-to-raster conversion tool was used to produce a raster map, which was classified using the quantile classification technique. 

Slope and elevation: Slope and elevation are two main factors used for earthquake potential zoning and earthquake-induced landslide susceptibility mapping [43]. Rough slopes can be observed in the hilly areas, where fault length and size are controlled by slope inclination [44]. These factors are quite important because hilly regions are seismically more active with complicated tectonics and structure as compared to plane lands. Therefore, these are two major input data used in earthquake probability estimation.

Fault density and distance from fault: Faults are the main source of earthquakes. High fault density denotes the areas with complex tectonics, thus increasing the probability of earthquakes [43]. The complex fault system or interconnected active faults can generate a high magnitude and number of events [45]. Distance from fault is significant because the earthquake potential zones are close to faults and decrease as the distance from fault increases [46]. In this study, major active faults were included for thematic layer preparation.

Magnitude, PGA, epicenter density, and distance from epicenter: Likelihood of a particular magnitude, location, depth, and PGA values depend on tectonic condition [43]. Epicenter density indicates the clustering of events that denotes the probable locations [46]. Epicenter density analysis could provide a way to focus on a structure’s riftogenesis study, a cluster of large earthquakes, and the large active fractures [43]. Such indicators are interlinked to each other because these can provide information on ground acceleration associated with several lithology types, maximum magnitude areas, and low seismic zones with the increase in distance from the earthquake source zone [43].

Lithology and amplification factor: Ground shaking depends on the amplification factor and rock types [43]. These are two major indicators because the amplification value of different rock types varies and could create either high or low ground shaking [43,46].

## 4. Methodology

### 4.1. CNN Architecture

**Figure 3:**
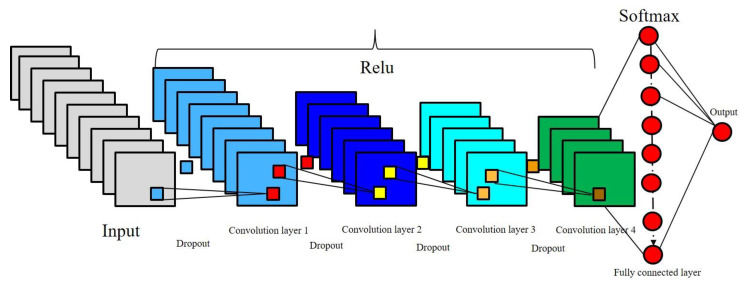
Architecture of the convolutional neural network (CNN) model for prediction of earthquake classification.

The architecture of CNN for earthquake prediction of classification consists of a sequential model with four convolutional layers, each of which is followed by a dropout and pooling layer. The details of the employed CNN layers are described below and presented in Figure 3: 

Input layer: Each layer is characterized by a 2D vector. An input sequence of n layers can be presented by concatenating the mathematical structure with multivalued points. The matrix $X\in R^{d\times n}$, where X is the network input [36].

Convolutional layer: A set of m convolutional filters is attached to this layer, where h is the length. $X_{\left[i:i+h\right]}$ indicates the concatenation of all data $X_{i.}$ to $X_{\left[i+h\right].}$ Therefore, the feature $C_{i}$ can be applied to a filter F using the following formula [37]:

(1) $$C_{i}:=\underset{k,j}{\sum }{\left(X_{\left[i:i+h\right]}\right)}_{k,j}.F_{k,j}$$

The concatenation of all data points in a layer describes the feature vector $C\in R^{n-h+1}$. Therefore, C vectors can be aggregated from all m filters into a feature map matrix $C\in R^{m\left(n-h+1\right)}$. During the training phase, the convolution filters within CNN are learned. A nonlinear ReLU activation function is applied to pass the output of the convolutional layer before entering a pooling layer.

Pooling layer: The aggregation of the input vectors occurred in a pooling layer, which is the maximum value over a set of non-overlapping intervals. The resulting pooled matrix can be expressed as $\;C\;\mathrm{pooled}\in R^{m\times \left(n-h+1/s\right)}$, where s is the length of each interval. However, when the stride value $s_{t}$ is observed in the case of overlapping intervals, the pooled matrix can be presented as $\;C\;\mathrm{pooled}\in R^{m\times \left(n-h+1-s/s_{t}\right)}$. The fraction result is rounded up or down, depending on the inclusion of the boundaries.

Hidden layer: A fully connected hidden layer is located at the end of the four convolutional layers. This layer computes the transformation  ∝(W×x+b), where the weight matrix is expressed as $W\in R^{m\times m}$, the bias can be estimated as $b\in R^{m}$, and the ReLU function is ∝. Consequently, the output can be presented as the $x\in R^{m}$ of this layer, which is similar to the mathematical structure of the input data [38].

Softmax: A softmax regression layer is connected to the outputs of the previous layer $x\in R^{m}$, which provides the largest probability values as ŷ ∈ [1, K] and can be expressed as:

(2) $$ŷ=\arg\;\max_{j}\;P\left(y=j|x,w,a\right)$$

(3) $$={\;\arg\;\max}_{j}\;\frac{e^{x_{\mathrm{wj}+\mathrm{aj}}^{t}}}{\sum_{k=1}^{k}e^{x_{\mathrm{wj}+\mathrm{aj}}^{t}}}$$

where $w_{j}$ indicates the weight vector of class j, from which the dot product can be generated with the input, and $a_{j}$ is the bias of class j. 

Optimization: The CNN parameters are learned using the Adam optimizer. The validation and parameters with the highest value should be computed and selected, respectively, at a fixed interval.

Loss estimation: The loss function is also called the cost function. This function measures the compatibility among output predictions and uses ground truth labels. In this model, a sparse categorical cross-entropy function is implemented as the loss function, which is beneficial for binary classification. However, the mean square error of the continuous values is applied to the regression. One of the hyperparameters is a loss function that can be determined on the basis of the given tasks.

Network parameters: The parameters learned during the training are as follows: θ={X, F1,  b1, F2, b2, W, a} (with X is the input data point matrix), where a d-dimensional vector can be found in each row for a specific layer; Fi and bi are used as the convolutional layer’s filter weights and biases, respectively; and W and *a* are the weight matrices in the softmax layer for the output classes.

### 4.2. Learning the Model Parameters and Performance

The parameters of the CNN model were learned through a three-phase procedure: (1) creation of data point embedding; (2) distant supervised phase, wherein the parameters are tuned by training, and; (3) final supervised phase, wherein the training is performed using the supervised training data [37]. In the distant supervised phase, heuristic techniques were used to reduce noise in the data points. This process can be regarded as a pretraining procedure to eliminate data noise and improve accuracy. The pretraining phase datasets do not necessarily overlap to generate the input embedding. CNN was trained for 1 epoch on this dataset before the final supervised training of 100 epochs. The weight of the data layers was updated in the distant and supervised training phases through backpropagation.

Accuracy is a simple measure of a classifier’s performance [36,37].

(4) $$\mathrm{Accuracy}=\frac{\mathrm{Number}\;\mathrm{of}\;\mathrm{correctly}\;\mathrm{labeled}\;\mathrm{samples}}{\mathrm{Number}\;\mathrm{of}\;\mathrm{all}\;\mathrm{testing}\;\mathrm{samples}}$$

The F1 score is computed from the precision and recall (i.e., the harmonic mean) of each class.

(5) $$F1={\left(\frac{{\mathrm{precision}}^{-1}+{\mathrm{recall}}^{-1}}{2}\right)}^{-1}$$

### 4.3. PGA, Source to Site Distance and Intensity Calculation

The earthquake catalog from USGS was used to estimate PGA based on the attenuation equation provided by Joyner and Boore [39]. The source to site distance was calculated using the epicentral distance provided in Equation (6). To estimate the intensity variation for the proposed study area, Equation (8) was used. Several attenuation equations have been proposed by various researchers, however, the current study applied the developed equation by Joyner and Boore [39]. Therefore, D value can be calculated using the formula written below:

(6) D=(E^2+7.3^2) ^0.5

where, E = Epicentral distance; and D = source to site distance.

According to Joyner and Boore [39], PGA can be calculated using the equation presented below:

(7) PGA=10^(0.249*M−Log(D)−0.00255*D−1.02,D=(E^2+7.3^2)^0.5

where, M = Earthquake magnitude, R= Radius from the epicenter to the location. There are several other attenuation relationships which could be implemented in probabilistic hazard analysis [40,41,42]. The most popular general equation of regression by Boore and Joyner [39] is used for regional and worldwide datasets for probabilistic analysis. Therefore, MMI (Modified Mercalli Intensity) could be estimated using the formula:

(8) MMI=1/0.3*(log10 (PGA*980)−0.014

where, the PGA unit is G (Gal).

Three main thematic layers were obtained such as MMI variation, PGA variation, and lithology with an amplification factor map using GIS along with several other layers from the same dataset. Firstly, various attributes of earthquakes were investigated in detail as per the requirement of earthquake probability assessment. Secondly, PGA and MMI were calculated based on the employed attenuation laws and implemented in GIS. PGA density map and intensity variation layers were utilized in earthquake probability mapping and hazard estimation. Several lithological units were extracted from the geological map and assigned amplification factor values to each unit to generate another thematic layer. The detailed information regarding the implementation of the CNN model and attenuation laws to generate the earthquake probability map was described in Section 5.

## 5. CNN Model Implementation for Prediction and Probability Mapping

**Figure 4:**
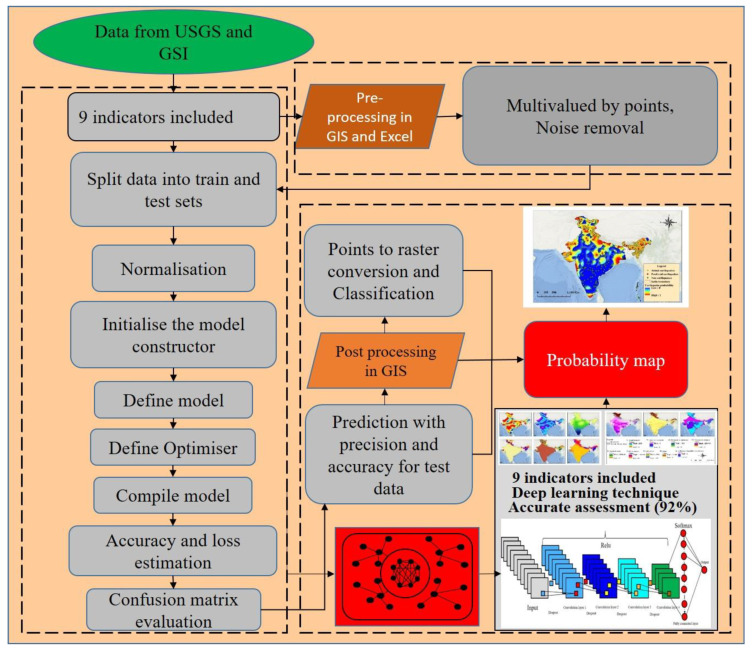
Methodological flowchart of the CNN model for earthquake probability assessment.

The detailed methodological flowchart of the employed model is shown in Figure 4. The implemented supervised classifier was trained using 75% of the spectrograms (training set) on a random subset, and performance was evaluated in terms of two-class classification. The trained dataset comprised 500 samples, with 350 and 150 samples used for training and validation, respectively. Once the model achieves good accuracy, the model is considered as well-trained. The selected datasets (20 million points) for testing all of India depend on the training sets that were used for probability assessment. 

In the first step, a comprehensive earthquake catalog was collected from USGS. Then, several random non-earthquake points were created to train the CNN classifier using GIS. Nine different indicators were used to extract multiple values by points and by keeping earthquake and non-earthquake as the target (Figure 4). In the second step, A CNN model was designed for earthquake prediction of two classes (0,1) that can continuously scan through several input indicators and convolutional layers and classify earthquake (1) and non-earthquake (0) values. After inspecting and discarding irrelevant data, the final dataset contained 250 earthquakes during the training period. The Adam optimizer was utilized to optimize the model, and then batch size (100), validation split (0.2), and verbose (1) were applied to avoid overfitting epochs (100). A total of 123,002 parameters were collected during training, all of which were trainable. The classifier predicted the target as 0 and 1 in the training and testing datasets for 500 points, respectively. Among the 500 points, 250 points were earthquakes and the rest were non-earthquakes. In the third step, post-processing was conducted to convert the pixels to raster to generate the probability map.

**Figure 5:**
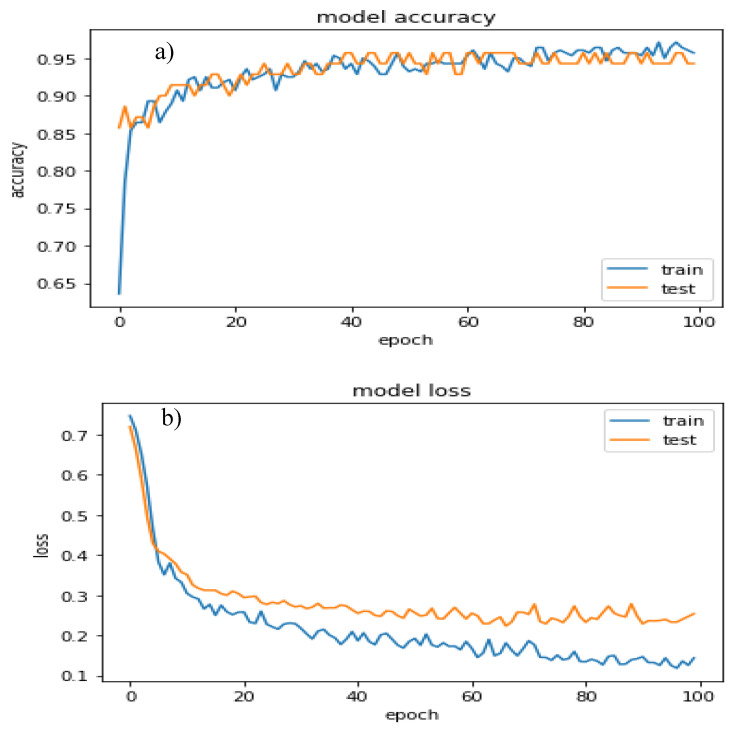
(**a**) Accuracy and (**b**) loss estimation of the CNN model for earthquake prediction.

The spatial layers generated for probability mapping were elevation, slope, lithology with an amplification factor, magnitude density, PGA density, depth density, distance from epicenter, distance from fault, and fault density (Table 1). Training was performed by splitting the data into training (75%) and testing sets (25%), normalizing and defining the variables, constructing and optimizing the model, and compiling the results for loss estimation [38]. Highly accurate prediction values can be obtained by applying the testing set. In this study, the number of earthquakes was determined by filtering the magnitude of quakes to higher than 4.5 within the period of 1900–2019 because low-magnitude events can be attributed to human activities and may not be destructive. There is no problem with the number of indicators to be used in a deep learning model. It has the capacity to run a huge number of indicators. However, model accuracy depends on the importance and weights of the factors. Therefore, several authors achieved low accuracy by applying unrelated factors in previous studies [38,39,40,41,42,43,44]. Therefore, these studies achieved low accuracy due to data heterogeneity and unrelated factors, which were removed from the study, applied nine major and useful factors, and described the importance of each factor in Table 1. Therefore, several layers from the study were removed such as aspect, hill shade, epicenter density, etc. This model was a predictive analysis-based probability assessment, wherein two classes were predicted. Then, the values for each pixel in the study area were predicted using the testing dataset. Each pixel value was converted into pixels using GIS to generate a probability map. The value for the probability map varies from 0 to 1. As probability varies between 0 and 1, then earthquakes (1) and non-earthquakes (0), while the pixels are not falling within these two classes, have a value between 0 to 1. Therefore, the map presented below is an unclassified probability map showing earthquakes and non-earthquakes. However, this model attempts to learn the associated general characteristics of earthquake and non-earthquake parameters from the indicators. Given that some thematic layers were prepared through GIS digitization, irrelevant pixel values originated can affect model performance. However, network performance can be further improved by applying all major indicators and noise removal techniques. Model performance was presented through graphs. Figure 5 shows the estimated loss and accuracy, and Table 2 lists the parameter details.

## 6. Results

### 6.1. CNN Classification and Bi-histogram Results

**Figure 6:**
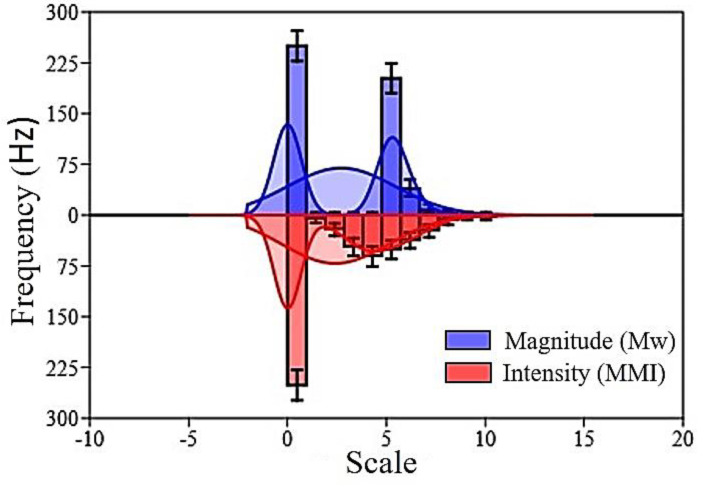
Bi-histogram of magnitude and intensity comparison using frequency that is used for probability assessment (Mw: moment magnitude, MMI: modified Mercalli intensity scale).

Major earthquake scenarios experienced in the past 200 years in the Indian subcontinent with magnitudes 7.0 to 8.0 and above were also included in the current study. Bi-histogram provides a relationship between magnitude, intensity, and frequency (Figure 6). In total, 11 bins formed for both the variables. Normal fit for both the magnitude and intensity shows the mirror reflection of each other. However, the kernel density of both variables does not project mirror reflection. Therefore, it reflects that the frequency varies for both the variables differently. The conversion of magnitude to intensity depends on the source-site distance. If the distance is less, then intensity could be higher for low magnitude events and extreme for high magnitude earthquakes. Similarly, if the distance is huge, then intensity could be lower for high magnitude events and negligible for low magnitude earthquakes. Therefore, the intensity values obtained in the current study applied to produce thematic layers as an indicator to generate a hazard map in the future.

The strong quakes that happened in India are the source locations that can produce future events in accordance with active faults. Previous quakes, such as Kutch (Mw = 8.3), Assam (Mw = 8.7), Kangra (Mw = 8.1), Bihar–Nepal (Mw = 8.3), and Assam (Mw = 8.6), are moving closer to the high hazard zones. Training is a major component of the CNN model to achieve an accurate result. Therefore, training and testing accuracies were calculated after the performance evaluation of the proposed CNN framework, reaching 96% and 92%, respectively. The estimated loss from this model was compared against the epochs. The obtained baseline error was 7.95%, and the final accuracy was 92.05%. The confusion matrix (Table 3) and the classification report (Table 4) targeting two classes presented, respectively. The false negatives in the confusion matrix did not follow any patterns. The result shows that the precision, recall, F1 score, and support for non-earthquake (0) and earthquake (1) classes were 0.98, 0.85, 0.91, and 71 and 0.88, 0.99, 0.93, and 80, respectively. The micro average and weighted average were also calculated and summarized in Table 4.

The CNN model’s accuracy reached 92.05% for the testing dataset. Table 3 and Table 4 present the details of the accuracy, error, and confusion matrices. The loss was estimated and plotted against the epochs. Loss decreases with an increase in the number of epochs. The predicted earthquakes were classified under the high- to very-high-hazard zones, indicating that the results are correct and useful.

### 6.2. Probability Mapping 

**Figure 7:**
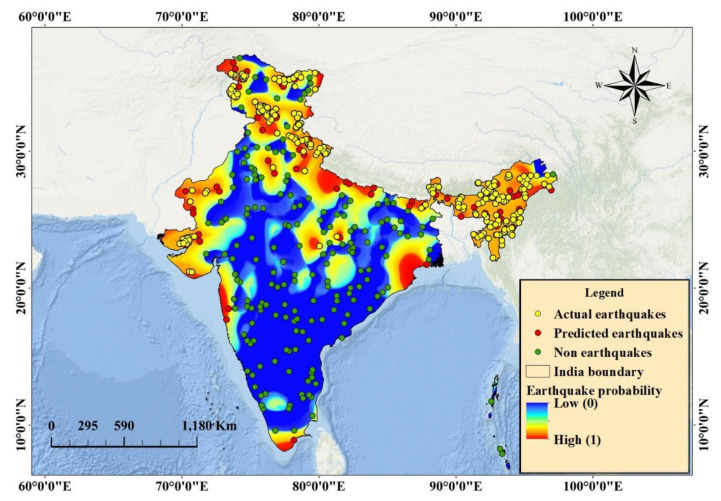
Earthquake probability map.

**Figure 8:**
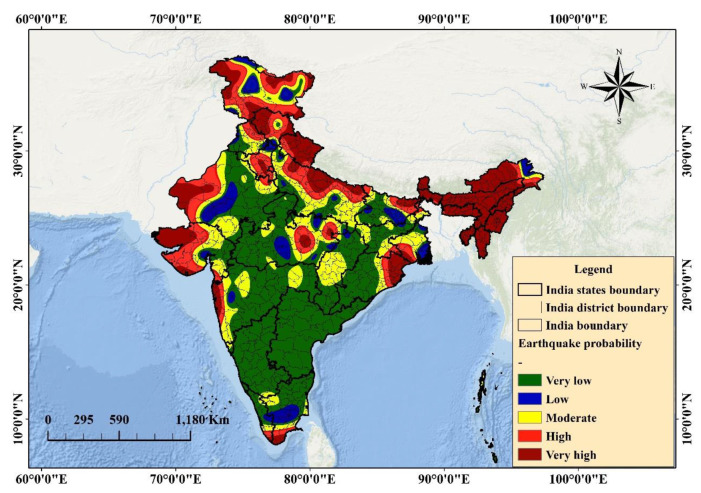
Classified earthquake probability map of India.

The probability zones of earthquake-prone areas were mapped for the Indian subcontinent. The probability map was obtained with values varying from 0 (no-earthquake) to 1 (earthquake) (Figure 7), and then presented through the classification of the probability (Figure 8). Five susceptible ranks were selected on the basis of the quantile classification technique to evaluate the probable areas, all of which fall within the ranks. However, the weight of the classes varies from very-high to very-low (Figure 8). The very-high-probability zone involves more than 200 events and can be found within the vicinity of active fault locations in the Himalayas. Very-high- to high-probability areas involve a source of strong vibration zones with high fault density, magnitude, and PGA values. The probable areas were determined quantitatively. 

**Figure 9:**
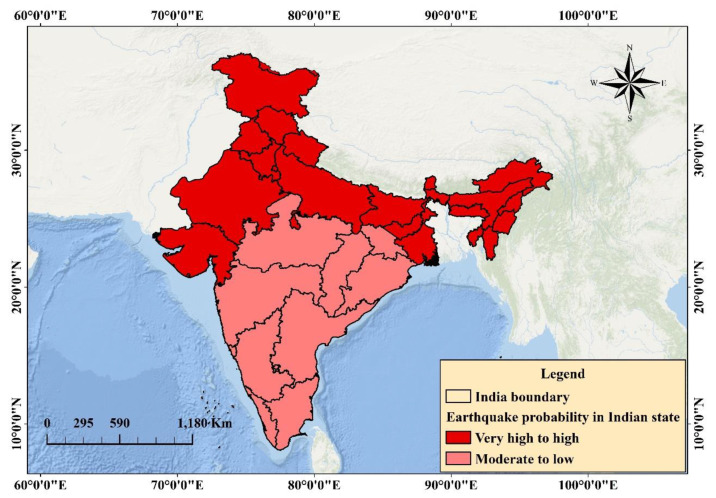
Two class-based scheme earthquake probability in all the states of India.

Table 5 presents the percentage of each zone, including the area (km^2^) contributing to active tectonics. The maximum area is within the very-low-probability zone (approximately 1,776,265 km^2^), followed by the low-probability (139,123 km^2^), moderate-probability (378,887.6 km^2^), high-probability (591,240.5 km^2^), and very-high-probability (712,375 km^2^) zones. Very-high- to very-low-probability areas comprise approximately 19.8%, 16.43%, 10.53%, 3.87%, and 49.37% of the aforementioned zones, respectively. A two class-based probability map was developed and presented in Figure 9. Several states that fall under very-high- to high-probability zones include Jammu, Kashmir, Northern Bihar, Chandigarh, Delhi, Haryana, Central Madhya Pradesh, Himachal Pradesh, Panjab, Gujrat, Rajasthan, Uttar Pradesh, Uttaranchal, Maharashtra, and northeastern states, where the total population comprises approximately half of India’s population based on the current census data (Table 5). The states that fall under the moderate- to very-low-probability zones are Tamilnadu, Kerala, Odisha, West Bengal, Chhattisgarh, Andhra Pradesh, Karnataka, the western part of Madhya Pradesh, Eastern Rajasthan, Central Bihar, Jharkhand, and some North Indian states.

Approximately 92% of predicted earthquake classes fall within the Himalayan zones (high- to very-high-hazard zones). These probable zones provide a holistic view, which is an integration of several parameters without considering a particular context of the scenario. This finding explains the severity of earthquake hazards that can originate from these regions in the future and provides an alarming view to local and national authorities with respect to necessary preparations for the worst situations with a gradual time course. 

### 6.3. Result Validation

The validation of results was conducted using a published seismotectonic zonation map and experts’ feedback. Sitharam et al. [26] reported the estimation of earthquake hazards at the surface level by applying necessary amplification factors with respect to several site classes based on the *V*_S30_ values obtained from the topographic gradient. They obtained a PHA at the surface level, wherein the return periods are 475 and 2475 years (presented in contour maps). 

Vipin et al. [35] estimated a seismic hazard in South India by implementing a probabilistic seismic hazard analysis. The seismic hazard map of India produced using GSI and the old seismic micro-zonation map of India show that the developed probability map is accurate and can be used in future studies on hazard and risk assessments. Five experts’ opinions were also considered to check the quality of the generated map (Table 6). Out of the five experts, four were highly satisfied (80%) with the obtained results and one was satisfied (20%).

## 7. Discussion

The associated earthquake probability with peninsular India was evaluated using a CNN model that was described in the implementation section of this paper. While integrating uncertainties associated with various modeling parameters, the most recent knowledge on seismic activity in the Indian subcontinent was applied to estimate probability. Uncertainties related to data that can be linked to a catalog or existing probable factors or other parameters involved in hazard analysis. The discussion part describes the comparison of old probabilistic results and seismic zoning maps with the obtained earthquake probability map. Most traditional techniques are poorly designed for low-magnitude events and do not record seismicity [47]. The objective of the current study is to develop a probability assessment model for independent earthquakes based on several indicators. A deep learning technique was proposed to achieve robust accuracy for probability assessment. However, CNN’s learning ability can vary with changes in the parameters [48]. Therefore, the sensitivity of the model was analyzed by applying changes to the convolutional layer filters and adopting various activation functions. Several significant changes were observed in the model’s performance. The model that exhibited the most satisfactory performance was selected. To avoid over-training, dropout rates were set as 0.2 and 0.1 for the convolutional and input layers, respectively, to facilitate the network in learning and performing a robust training process. The applied dropout rate exhibits the best trade-off between validation accuracy and training data misfit. 

Khattri et al. [49] developed the first probabilistic seismic hazard map of the Indian subcontinent. They divided the region into 24 seismic sources for hazard parameter estimation based on peak acceleration for 50 years, wherein the exceedance probability was 10%. However, the intensity values for hazard estimation were not used in the present study. Instead, an earthquake probability map based on predictive analysis was presented. Khattri et al. [49] reported that a maximum peak acceleration of 0.8 g was expected in the northeast region, and the value varied from 0.03 g to 0.04 g in the south, southeastern, and central parts. Similar values were observed for Northeast and Central India. On average, the PGA values of the Himalayan region varied from 0.5 to 1.01 (Figure 7). Bhatia et al. [50] developed another probabilistic seismic hazard map for India. The hazard level was estimated on the basis of the PGA values after locating 86 potential sources based on seismicity trends and major tectonic features. Joyner and Boore’s [51] attenuation equation was applied to this probabilistic assessment. Aman et al. [52] and Singh et al. [53] performed a probabilistic assessment in the Himalayan regions, and the two groups of researchers achieved similar results. Lyubushin and Parvez [54] applied a purely statistical procedure for probability assessment. However, the present study did not focus on hazards. 

**Figure 10:**
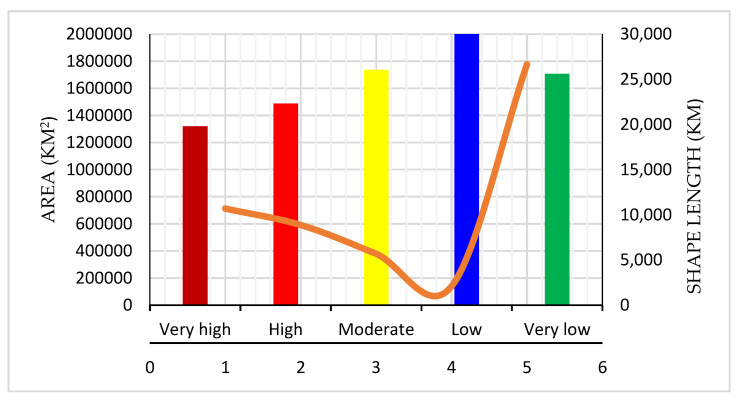
Graphical presentation of probability among classes, area, and distance.

The probability assessment was completed on the basis of nine factors (Table 1). Figure 8 presents the results of the predictive analysis-based probability assessment and the probability map. According to the literature, CNN is one of the most reliable models for probability assessment; ANN provides an accuracy of 84%. The proposed technique performs better than ANN prediction while significantly reducing processing time and improving efficiency. In addition to event prediction, the proposed approach provides the best estimates of probability from the events that already occurred. The plot of the probable classes (with area and shape length) explains the variation in the probability with respect to distance and area (Figure 10). 

The objective was to produce an earthquake probability map based on experienced events. In this study, the authors did not implement any technique to predict events based on magnitudes, intensity, time, or location. This study is a prediction of classification-based probability assessment; therefore, the areas close to the experienced events will be the probable areas for earthquakes. First, pixels close to the predicted classes can be interpolated to generate a probability map. Second, the entire area can be converted into points for testing using the same model to obtain the pixel values for each point and generate a probability map. Both techniques are effective; however; the second one is more accurate than the first one. In accordance with the old hazard map of India, the Himalayan region belongs to Zone V, which includes Gujarat State in the west, and some cities belong to Zone IV. However, in accordance with the seismic zoning map of India (BIS, 2002), a large part of the Narmada lineament region spreads up to the northern part of the Godavari graben (https://law.resource.org/pub/in/bis/S03/is.1893.1.2002.pdf). Therefore, the hazard estimated in this region is higher in contrast with the Indian Standard code. The current study of probability mapping is linked to the hazard map, which is under Zone IV but with low probability. Southern portions, including Tamil Nadu, are under passive margin and exhibit higher ground motion based on the hazard map (Zone III) while presenting low probability in accordance with the obtained map. The western portions of some states, such as Bengal and Orissa, and the western part of Madhya Pradesh, Chhattisgarh, Andhra Pradesh, and Karnataka are estimated as moderate to low probability and low hazard zones on the basis of the seismic zoning map (Figure 10). Therefore, these areas are considered the stable shield of peninsular India. However, the resulting probable areas are nearly similar to other parts of the hazard map of India. The resultant earthquake probability map indicates that priority should be focused on the Central Himalayas and Northwest, Northeast, and Shillong areas of the Indian subcontinent; because of the complicated tectonics and active faults in areas where no large earthquake has occurred since the 2011 Sikkim earthquake. Other priority areas experienced mild events such as Maharashtra, Kachchh region, cratonic regions, and rift basins in India that can be considered for future hazard and risk studies. A national database of active faults should be created, and event prediction should be initiated using GIS [55]. The database would enhance the geological and earthquake hazard-related issues under different tectonic settings using machine learning techniques [56,57].

## 8. Conclusions

On the basis of the recent advances in deep learning, a CNN model was proposed to achieve a robust prediction of experienced earthquake events to address the shortcomings of existing methods and develop a probability map for the Indian subcontinent. In the current study, the authors attempted to estimate the probable areas (spatial) for future earthquakes. The final probability map achieved an accuracy of 92.05%. Therefore, the proposed model is superior to traditional methods for earthquake probability assessment in terms of accuracy. From the results, very-high (712,375 km^2^) and high (591,240.5 km^2^) probability areas consist of 19.8% and 16.43% of the aforementioned zones, respectively. The limitation of this study includes the implementation of CNN, which is time- and data-consuming, particularly the training data. This study is limited to earthquake figure probability assessment. The geological and deep learning constraints presented in this research are insufficient for probability mapping. The detailed analysis of factors, including geophysical and precursor studies, could be enhanced to improve the accuracy of the probability assessment. Several assumptions were made and model parameter modification was performed during the entire process, such as the selection of earthquakes with more than 4.5 Mw, distance criteria, including maximum magnitude scenarios, removal of unwanted thematic layers, and the weighting scheme within the CNN model. The earthquake probability map presented in this study is intended to highlight nine influencing parameters that can be changed further with geophysical, geomorphologic, seismic, and field data. The probability map based on CNN can be useful for earthquake hazard and risk mapping, mitigation, land use planning, and other related applications. Understanding the relative importance of the applied factors is crucial for further studies. This study can further support hazard and mitigation strategies and proper planning for critical facilities, including hydropower projects, dams and tunnels, nuclear power plants, and other engineering structures. Source-specific paleoseismic parameters, geodetic considerations, and geophysical parameters can depict micro-level sources in future research. Accurately dealing with associated uncertainties can be another future focus to achieve a good assessment in the field of earthquake probability assessment. More research should be done to improve the seismic probability mapping using state-of-the-art machine learning approaches and advanced artificial intelligence techniques.

## Figures and Tables

**Table 1:** Earthquake probability parameters and their characteristics.

Parameters	Data Source	Resolution	Scale	Description
Slope Elevation	DEM (USGS) https://earthexplorer.usgs.gov/	30 m	1:250000	Derived from raster DEM
Fault density Distance from fault	Geological map of India, GSI			Derived from image digitization in ArcGIS
Magnitude density Epicenter density Distance from epicenter	USGS earthquake catalog (https://earthquake.usgs.gov)			Derived using Joyner and Boore (1981), Campbell (1981)
PGA density	USGS earthquake catalog			PGA can be derived using (MMI=1/0.3*(log10 (PGA*980)−0.014)
Lithology and amplification factor	Geological map of India, GSI (www.gsi.gov.in), (bhuvan.nrsc.gov.in), *(USGS World Geologic Map)*			Derived from image digitization in ArcGIS 1. Unknown:1 2. Hard rock:0.55 3. Soft rock:0.70 4. Medium soil:1 5. Soft soil:1.30

**Table 2:** CNN model parameters for prediction.

Layer (Type)	Output	Shape Parameter
dense_1 (Dense)	(None, 200)	2000
dropout_1	(None, 200)	0
dense_2 (Dense)	(None, 200)	40,200
dropout_2	(None, 200)	0
dense_3 (Dense)	(None, 200)	40,200
dropout_3	(None, 200)	0
dense_4 (Dense)	(None, 200)	40,200
dropout_4	(None, 200)	0
dense_4 (Dense)	(None, 2)	402
Input number of units = 9		
Output = 2		
Hidden units = 200		
Kernel regularizer = l2(0.0001)		
Activation = ‘relu’		
Activation = ‘softmax’		
Total params: 123,002		
Trainable params: 123,002		
Non-trainable params: 0		

**Table 3:** Confusion matrix for the prediction model.

		**Predicted**	
		**Positive**	**Negative**
Actual	**Positive**	60	11
	**Negative**	1	79

**Table 4:** Performance of the developed CNN model.

Classification Report	Precision	Recall	F1 Score	Support
**0**	0.98	0.85	0.91	71
**1**	0.88	0.99	0.93	80
**Micro average**	0.92	0.92	0.92	151
**Micro average**	0.93	0.92	0.92	151
**Weighted average**	0.93	0.92	0.92	151
**Prediction accuracy: 0.920530**				

**Table 5:** Earthquake probable areas in km^2^ and percentage.

Class No.	Probability Classes	Shape Length (km)	Area (km^2^)	Area (%)
1	Very-high	19,788.24	712,375	19.8
2	High	22,309.64	591,240.5	16.43
3	Moderate	26,041.08	37,8887.6	10.53
4	Low	30,004.07	139,123.1	3.87
5	Very-low	25,599.15	1,776,265	49.37
Total			3,597,891	100

**Table 6:** Experts’ profile and feedback on the probability results.

Category	No. of Experts	Profession	Specialization	Recruitment Process	Validation Criteria	Feedback
Researchers	5	Seismologist, geologist, hydrologist, GIS analyst, soil physicist, geotechnical researcher	Researcher on natural hazards using GIS and remote sensing, monitoring, mapping, GIS, artificial intelligence	Deep learning application to earthquake prediction Geological mapping with sensitivity Expert in local and regional earthquake probability and risk assessment Data analysis expertise Published good articles in high-impact journals	Probability determinants Representation of spatial maps Expected uncertainties Map validation using old maps by GSI Results Communicability Usefulness to land use planning Benefit to local people	Four experts are highly satisfied (80%) One expert is satisfied (20%)
